# A Recent Overview of Producers and Important Dietary Sources of Aflatoxins

**DOI:** 10.3390/toxins13030186

**Published:** 2021-03-03

**Authors:** Darina Pickova, Vladimir Ostry, Frantisek Malir

**Affiliations:** 1 Department of Biology, Faculty of Science, University of Hradec Kralove, Rokitanskeho 62, CZ-50003 Hradec Kralove, Czech Republic; ostry@chpr.szu.cz (V.O.); frantisek.malir@uhk.cz (F.M.); 2 Center for Health, Nutrition and Food in Brno, National Institute of Public Health in Prague, Palackeho 3a, CZ-61242 Brno, Czech Republic

**Keywords:** aflatoxigenic microfungi, aflatoxins, food

## Abstract

Aflatoxins (AFs) are some of the most agriculturally important and harmful mycotoxins. At least 20 AFs have been identified to this date. Aflatoxin B_1_ (AFB_1_), the most potent fungal toxin, can cause toxicity in many species, including humans. AFs are produced by 22 species of *Aspergillus* section *Flavi*, 4 species of *A.* section *Nidulantes,* and 2 species *of A.* section *Ochraceorosei*. The most important and well-known AF-producing species of section *Flavi* are *Aspergillus flavus*, *A. parasiticus*, and *A. nomius*. AFs contaminate a wide range of crops (mainly groundnuts, pistachio nuts, dried figs, hazelnuts, spices, almonds, rice, melon seeds, Brazil nuts, and maize). Foods of animal origin (milk and animal tissues) are less likely contributors to human AF exposure. Despite the efforts to mitigate the AF concentrations in foods, and thus enhance food safety, AFs continue to be present, even at high levels. AFs thus remain a current and continuously pressing problem in the world.

## 1. Introduction

Aflatoxins (AFs) are some of the most important and harmful mycotoxins. As of 2020, 60 years have already passed since their discovery. AFs are one of the five agriculturally most important mycotoxins [1,2,3,4]. Chemically, the AFs are difuranocoumarin derivatives with a bifuran group attached to the coumarin nucleus and a pentanone ring (in the case of aflatoxin AFBs) or a lactone ring (in case of aflatoxin AFGs) [5]. There are more than 20 known AFs, but the most common are aflatoxin B_1_ (AFB_1_) (PubChem CID: 186907), aflatoxin B_2_ (AFB_2_) (PubChem CID: 2724360), aflatoxin G_1_ (AFG_1_) (PubChem CID: 14421), and aflatoxin G_2_ (AFG_2_) (PubChem CID: 2724362) (PubChem, 2020), from which AFB_1_ is the major representative in food crops [6]. Aflatoxin M_1_ (AFM_1_) (PubChem CID: 15558498) and M_2_ (AFM_2_) (PubChem CID: 10903619) are the hydroxylated metabolites of AFB_1_ and AFB_2_ [7,8,9]_._

AFs are acutely toxic, hepatotoxic, immunosuppressive, mutagenic, teratogenic, and carcinogenic compounds [10,11,12,13,14]. The International Agency for Research on Cancer (IARC) evaluated the carcinogenicity of naturally occurring AFs (AFB_1_, AFB_2_, AFG_1,_ and AFG_2_) for humans as Group 1 ”carcinogenic to humans” in 1987 [10,15], and re-evaluated in 2012 [16,17]. AFM_1_ is often misclassified in the literature as Group 1; however, it was classified as Group 2B “possibly carcinogenic to humans” in 1993 [1] and has not been re-evaluated since. For these reasons, AFs need to be monitored and their concentrations in food should be kept at the lowest possible levels.

While acute exposure to a high dose can result in vomiting, abdominal pain, and even death, chronic exposure to low doses may lead to liver cancer [18,19], which is generally considered to be the most significant impact of AFs on human health [10,20]. According to the latest data from the Global Cancer Observatory, liver cancer is the sixth most common cancer for both sexes of all ages, with a total of 905,677 new cases estimated in 2020 [21]. It has been estimated that AFs contribute to 4.6% to 28.2% of all global hepatocellular carcinomas [22].

Nowadays, AFs are of great interest as they are one of the most serious contaminants that can significantly affect the food chain. Humans, at the top of the food chain, often consume contaminated foodstuffs of both plant and animal origins. Besides human health, food insecurity caused by AFs contamination can also affect humanity at the social, political, and economic levels [23].

Therefore, in this article, attention is paid to AFs in terms of AF producers and the occurrence of AFs in foods around the world.

## 2. Producers of Aflatoxins

To date, AFs are produced by 28 species of the genus *Aspergillus*. *Aspergillus* subgenus *Circumdati* section *Flavi* contains some of the most important species in the genus, which usually produce AFs [24,25,26].

The accurate identification of *Aspergillus* section *Flavi* requires a polyphasic approach that includes the morphological characters (the microscopic structures, such as the uni- or biseriate conidial heads, the production of dark-colored sclerotia by certain species, and yellow-green to brown shades of conidia), and the chemical (extrolite data) and molecular (partial sequences of calmodulin, β-tubulin, and internal transcribed spacer region) approaches, as these species are closely related and could not be easily distinguished by morphological characteristics alone [24,25,26].

*Aspergillus* section *Flavi* currently contains a total of 34 species in 8 clades: the *Aspergillus alliaceus*-, *A. avenaceus*-, *A. bertholletius*-, *A. coremiiformis*-, *A. flavus*-, *A. leporis*-, *A. nomius*-, and *A. tamarii*-clade [24,25,26,27]. The three new clades *A. texensis*-, *A. agricola*-, and *A. toxicus*-clade with three species were presented in the year 2020 [28,29].

Table 1 gives an overview of the current identity of *Aspergillus* species from *Aspergillus* section *Flavi* as AF producers focus on foodstuffs [24,25,26,27,28,29,30].

The most important and most well-known AF-producing species of section *Flavi* in foodstuffs are *Aspergillus flavus* [31,32], *A. parasiticus* [33,34,35], and *A. nomius* [36,37]. While *Aspergillus flavus* produces AFB_1_ and AFB_2_, *A. parasiticus* and *A. nomius* can produce AFB_1_, AFB_2_, AFG_1_, and AFG_2_.

*Aspergillus minisclerotigenes* and *A. parvisclerotigenes* also belong to section *Flavi*. Both have morphological and physiological similarities to *A. flavus*; however, they produce more but smaller sclerotia. In contrast to *A. flavus*, this is usually coupled with a high and consistent production of both the B and G type of AFs [24].

In addition to *Aspergillus flavus*, four other *A.* species (*A. agricola*, *A. pseudotamarii*, *A. togoensis,* and *A. toxicus*) only produce AFB_1_ and AFB_2_. Seventeen other *Aspergillus* species can produce AFB_1_, AFB_2_, AFG_1_, and AFG_2._ It is generally accepted that *A. flavus* is unable to produce AFs type G, but it is also reported that some Korean strains are capable of producing both AFG_1_ and AFG_2_ [25]_._ However, some *Aspergillus* species from *Aspergillus* section *Nidulantes* [38] or *Aspergillus* section *Ochraceorosei* [32,39] can also produce AFs.

The identification of *Aspergillus* section *Nidulantes* requires a polyphasic approach which includes the morphological characters (the microscopic structures such as the color, shape, size, and ornamentation of ascospores, the shape and size of conidia and vesicles, and growth temperatures), and the chemical (extrolite data) and molecular (internal transcribed spacer region, partial β-tubulin, calmodulin, and RNA polymerase II the second largest subunit (RPB2) gene sequences) approaches [38]. Based on this polyphasic approach, *Aspergillus* section *Nidulantes* was subdivided into 7 clades and 65 species [38]. The majority of section *Nidulantes* species can produce a sexual state, and those species were, in the dual name nomenclature system, assigned to the genus *Emericella*. Because of the adoption of the “one fungus: one name” nomenclatural system, all *Emericella* species were transferred to *Aspergillus* [40]. AFB_1_ was produced by four species: *Aspergillus astellatus* [41], *A. miraensis* [42,43], *A. olivicola* [44], and *A. venezuelensis* [45]. *Aspergillus ochraceoroseus* and *A. rambellii* belong to section *Ochraceorosei* [32]. *A. ochraceoroseus* produce AFB_1_ [11,39,46,47], and *A. rambellii* also produce AFB_1_ [32,39]_._

With the development of modern molecular biological and chromatographic methods, other new AF producers will certainly be identified soon and bring new research to this area.

## 3. Aflatoxin Occurrence in Foods

The contamination of foods with AFs, like with other mycotoxins, has become a global problem [48]. For several years, a statement claiming that a total amount of 25% of the world’s crops are affected by molds and mycotoxins, supposedly estimated by the Food and Agriculture Organization (FAO), has been circulating worldwide [12,49]. However, this estimation has been challenged in the most recent studies dealing with the background of this matter, as this statement was not possible to trace back, since even FAO experts were not able to do so [50]. On the basis of an extensive study by the BIOMIN Company in 2004–2011, 72% of samples of feed (mainly maize, wheat, barley, and silage) and feed raw materials (especially for swine, poultry, and cows) from all over the world, but mainly from Asia (40%) and Europe (38%), contained a detectable amount of at least one mycotoxin including AFs. Moreover, a co-occurrence of two or more mycotoxins was confirmed in 38% of samples [51], and of course, AFs can interact in synergy with other mycotoxins. This fact is alarming since the major intake of mycotoxins into human organisms is usually due to dietary exposure [52], and even a low concentration of AFs is hazardous for humans [53].

In general, inappropriate storage is considered a major cause of foods contamination with mycotoxins—especially in developing countries [54,55], in which approximately 20% of the global volume of potentially highly contaminated commodities originate [56]. In some cases, contamination of crops with mycotoxins may already occur in the field due to stress factors such as insects or drought that facilitate the contamination [57]. Climate conditions, such as high temperatures, heavy rainfalls, and high relative humidity, are likely to contribute to crop contamination as well, as they make plants more susceptible to fungal, and thus mycotoxin, contamination [58,59]. Contamination during transport and processing is also possible [23]. Good agricultural, manufacturing, and hygienic practices, good plant disease management, and adequate storage conditions can limit mycotoxin levels in the food chain, yet these practices do not eliminate mycotoxins completely [60,61].

Fortunately, some contamination-reducing chemical (ammonization, hydrogen peroxide, sodium bisulfate, organic acids, ozone, and plant extracts), physical (separation, solvent extraction, mineral adsorbents, heating, extrusion, microwaving, irradiation, and UV radiation) and biological (enzymes, bacterial cells, yeast cells, and non-toxigenic strains) technologies have been developed to enhance food safety [20,23]. However, the European Union legislation, in Section 2 of the Annex “Mycotoxins”, does not allow any foods contaminated with mycotoxins to be detoxified by the chemical approach [62]. Moreover, foods treated by sorting or other physical means must not be mixed with foods intended for direct human consumption nor with foods intended to be used as food ingredients [62]. Biological control, depending on the competition between non-toxigenic and toxigenic strains, is the most commonly used method, especially in countries where AFs pose a significant threat [63]. For example, a product *Aflasafe*^TM^ has begun to be applied to reduce AFs with an average efficiency of 99% (76%–100%) in maize and groundnuts [64,65,66]. The principle of its use lies in the contamination of crops with non-toxigenic strains before they are contaminated by toxigenic strains of *Aspergillus flavus. Aflasafe*^TM^ is a relatively cheap and easy-to-apply product that ensures a long-lasting reduction of AFs (up to consumption level) [64].

AFs contaminate a wide range of foods of both plant and animal origin. AFB_1_, AFB_2_, AFG_1_, and AFG_2_ are major contaminants in commodities of plant origin, mainly groundnuts, tree nuts, spices, seeds, dry fruits, and cereals [67,68,69]. The daily intake of AFs at the level of nanograms to micrograms per person per day is mainly achieved through the consumption of contaminated maize and groundnuts [70]. Animal products are less likely substrates for AF producers; however, the metabolites AFM_1_ and AFM_2_ are typical in milk, including human breast milk [71,72], and dairy products of lactating ruminants that have been fed with contaminated feed (carry-over to dairy milk) [73,74,75,76]. AFM_1_ has also been detected in cheese worldwide [77,78,79] and AFs (in low concentrations) have been reported to occur in certain products of animal origin, such as meat and meat products, or eggs, etc. (carry-over of AFs in products of animal origin) [74].

Drought periods combined with high temperatures significantly increase AF production in the fields [80]. It has been estimated that at least 4.5 billion people worldwide are chronically exposed to AFs from foods, especially in “hot zones” in the regions situated between 40° N and 40° S latitude [81]. Climate change and the trend of global warming may lead to an increased occurrence of mycotoxins, for the production of which higher temperatures are needed, and the same goes for AFs [82]. This might be the case in Northern [82] or Western [83] Europe, for example, where AFB_1_ contamination of maize was recently observed [84]. It should be emphasized that even in the current modern age, cases of acute aflatoxicosis leading to human death may occur due to climate change [85]. Climate change is dealt with in more detail in the Special Issue of Toxins entitled “Mycotoxins in Relation to Climate Change”.

On the other hand, it is known that the AFs belong to the dominant mycotoxins in the African and Asian continents, as well as North and South America and the Australian continent [86]. Additionally, despite all efforts to mitigate AFs in foods, there are still cases of high AF concentrations in foods. Therefore, to enhance food safety, there is a global need for regulatory limits and food contaminant monitoring tools.

### 3.1. The Occurrence of Aflatoxins in Food in the African Continent

In African countries, maize and groundnuts represent the largest exposure to AFs [87,88], where maize is a staple crop for the majority of the African population [88,89]. The case of highly contaminated (1–46, 400 µg/kg) maize in Kenya in 2004, associated with 125 human deaths, is historically relevant [85].

There are still cases of concentrations exceeding the limits set in many countries. Recently, high concentrations of AFs in maize grains of up to 9091.8 µg/kg (AFB_1_) were found in Kenya [89], up to 3760 µg/kg for total AFs (where AFT is the sum of AFB_1_, AFB_2_, AFG_1_, and AFG_2_) in Uganda [90], up to 2806.5 µg/kg (AFT) in the Democratic Republic of Congo [91], up to 1460 µg/kg (AFT) in Nigeria [88], up to 945 µg/kg (AFT) in Ghana [92], and up to 107.6 µg/kg (AFT) in Zambia [93].

### 3.2. The Occurrence of Aflatoxins in Food in the Asian Continent

Practically all tropical countries face the problem of AFs [94]. The climate of Asian countries is very favorable for AF-producing microfungi [95], especially when it comes to commodities such as cereals—mainly maize and rice, cereal products, beans, groundnuts, and other oily products—which is alarming, as cereals and groundnuts are considered major items in the Asian diet [94].

AFs were found in maize in concentrations of up to 1572 µg/kg (AFB_1_) in Vietnam [96]. However, in Asia, rice is the most important crop in terms of its consumption [55,97], and especially production, as approximately 90% of the world’s rice is produced in Asia, of which nearly two-thirds are produced by China, India, and Indonesia [98]. High concentrations of AFs in rice have been reported in many scientific studies. In the case of AFB_1_, reported concentrations reached up to 361.0 µg/kg in India [99], up to 185.0 µg/kg in Sri Lanka [100], and up to 26.6 µg/kg in Thailand [101]. In the case of AFT concentrations, they were found to reach up to 96.3 µg/kg in Malaysia [102], up to 77.8 µg/kg in Vietnam [55], up to 21.4 µg/kg in Turkey [103], and up to 21.0 µg/kg in China [104].

### 3.3. The Occurrence of Aflatoxins in Food in the American Continent

America is the largest producer of maize (565 million tons in 2019; 49.2% of world production). The United States, Brazil, Argentina, and Mexico belong to the top 10 producers worldwide [98]. Alongside sub-Saharan Africa and Southeast Asia, maize is a staple food in Latin America [105], especially in Guatemala [106] and Mexico [107].

However, concentrations of AFB_1_ of up to 2656 µg/kg were observed in maize in Guatemala and are potentially high throughout the rest of Central America and Mexico [106]. Lower concentrations of up to 282.5 µg/kg (AFB_1_) and 303.9 µg/kg (AFT) were detected in maize kernels in South Haiti [108], and concentrations of up to 49.9 µg/kg (AFT) were found in Brazil [109]. Processed maize products are also contaminated with AFs. For example, tortillas and popcorn have been reported to be contaminated with up to 287.23 µg/kg (AFB_1_) [110] and up to 120 µg/kg (AFT) [111], respectively, in Mexico.

Of course, the problem is not only maize as a staple food, as high levels of AFs are also found in other local commodities, including up to 33.3 µg/kg (AFT) in nuts, up to 176.4 µg/kg (AFT) in *Capsicum* spices in Chile [112], and up to 70.9 µg/kg (AFT) in the case of Brazilian rice [113].

### 3.4. The Occurrence of Aflatoxins in Food in the Australian Continent

In Australia, hot and dry conditions typical for the arid and semi-arid areas covering much of the continent are the main stress factors that allow for the contamination of crops with AFs. This represents a major problem in Australia in terms of peanut degradation [114,115]. The occurrence of AFs is not quite as common in Australian maize [116], and when it occurs, it is in low or moderate concentrations [117] for unknown reasons [115]. Nevertheless, maize is only a small part of the human and animal diet in Australia [115].

The occurrence of AFs in Australian maize is usually in the range of 1–5 µg/kg, but can also occasionally reach higher concentrations of up to 200 µg/kg [118]. However, higher concentrations of AFT in maize (up to 311.1 µg/kg), and also in peanuts (up to 384.8 µg/kg), sorghum (up to 138.3 µg/kg), and wheat (up to 26.8 µg/kg), have been found in Australia [115,119,120,121].

### 3.5. Aflatoxin Regulations in the European Union and around the World

The discovery of AFs and their serious negative effects on human and animal health in the early 1960s led many countries in the world to establish certain regulations of mycotoxins in foods to protect consumers from the harmful effects caused by mycotoxins [122,123]. The first limit regulating mycotoxins, namely AFs, was set in the late 1960s, and by 2003 approximately 100 countries in the world had already regulated mycotoxins in foods [123]. Although the number of countries regulating mycotoxins in foods is increasing [123], most African countries and other developing countries lack regulations [92], as the compliance with the limits in developing countries would result in a shortage of food, and thus an increase in its price.

From the perspective of all mycotoxins, the regulations of AFB_1_, AFT, and AFM_1_ are the greatest concern of worldwide legislation [124]. The Codex Alimentarius specifies an AF maximum limit of 15 µg/kg (for almonds, hazelnuts, Brazil nuts, peanuts, and pistachio nuts for further processing) and 10 µg/kg (for almonds, Brazil nuts, hazelnuts, and pistachio nuts for direct consumption and dried figs), and AFM_1_ maximum limit of 0.5 µg/kg for milk [125]. However, the maximum levels of AFs in foods vary throughout different countries depending on the type of product and also on the import/export regime [69].

The European Union (EU) has one of the most comprehensive and strictest regulations on AF levels, set by the commission regulation 1881/2006 [62], and later on by its amending supplement 165/2010 [126], that are binding upon the 27 member states of the EU. These levels are in ranges 0.1–12 µg/kg, 4–15 µg/kg, and 0.025–0.05 µg/kg for AFB_1_, AFT, and AFM_1_, respectively, in the case of various foods [62,126].

For comparison with other countries, the maximum limit/regulatory limit/action level (or the range) for AFB_1_ has been set at 30 µg/kg in India, at 20 µg/kg in the Philippines, at 15–20 µg/kg in Indonesia [127], at 0.5–20 µg/kg in China [128], at 5–10 µg/kg in Japan, and at 0.1–10 µg/kg in Korea [127].

The maximum/action limit (or the range) for AFT has been set at 20–35 µg/kg in Indonesia [127]; at 5–15 µg/kg in Malaysia [129]; at 30 µg/kg in Sri Lanka [127]; at 20 µg/kg in the United States [130,131,132,133], Thailand, the Philippines [127], and Nigeria [134]; at 15–20 µg/kg in Hong Kong [127]; at 1–20 in Brazil µg/kg [135]; at 15 µg/kg in Canada [136], Korea [69,127], Australia [137], and Zimbabwe [134]; at 0–15 µg/kg in Taiwan, at 10 µg/kg in Japan, Vietnam [69,127], Kenya, Mozambique, South Africa, and Uganda [134]; and at 5 µg/kg in Singapore [127].

If a country has any regulation on AFM_1_ in milk or dairy products, it is usually set at 0.5 µg/kg [128,135,138,139], which is in line with the Codex Alimentarius. However, in the EU legislation, the AFM_1_ maximum limits (0.025–0.05 µg/kg) are 10–20 times lower compared to the Codex Alimentarius (0.5 µg/kg) [62,125].

### 3.6. The Occurrence of Aflatoxins Based on Data by INFOSAN (2016–2020)

The International Food Safety Authorities Network (INFOSAN) is a global information network jointly managed by the World Health Organization (WHO) and the FAO [140]. The INFOSAN has facilitated urgent international communication during food safety emergencies between more than 600 members from 188 of the 194 FAO and WHO member states since 2004. The INFOSAN aims to reduce the incidence of foodborne diseases that have a significant impact on public health and international trade [140,141]. Regarding AFs, only two cases, both of which concerned maize in Tanzania, were reported in 2016 and 2017 [142]. There have been no reports on AFs in foods since.

### 3.7. The Occurrence of Aflatoxins in Food Based on Data by RASFF (2015–2020)

The Rapid Alert System for Food and Feed (RASFF) is an important warning system for food and feed safety from the perspective of the EU countries [143]. Regarding the number of notifications reported by RASFF in 2015-2020, most mycotoxin notifications were related to AFs (approximately 88%), of which most were of the food category (approximately 94%) and less were of the feed category (approximately 6%), as shown in Table 2 [144].

Based on data from the last years (2015–2020), the vast majority of notified products contaminated with AFs belong to the “nuts, nut products, and seeds” category, followed behind by “fruits and vegetables”, “herbs and spices”, “cereals and bakery products”, and others. Namely, the most often notified foods are, in descending order, groundnuts, pistachio nuts, dried figs, hazelnuts, spices, almonds, rice, melon seeds, Brazil nuts, and maize [144].

Throughout the years 2015–2020, cases of very high concentrations of AFs in foods were notified. Based on these “high-level” notifications, groundnuts, pistachio nuts, almonds, dried figs, hazelnuts, chilies, melon seeds, and apricot kernels appear to be highly contaminated (with the maximum concentration of AFB_1_ or AFT exceeding 1000 µg/kg). Spices (other than chilies), tiger nuts, Brazil nuts, rice, pecan nuts, walnuts, and maize represent the less contaminated foods [144]. There is a concern for the development of aflatoxicosis associated with the consumption of foods with an AF concentration of at least 1000 µg/kg [145]. This implies that the group of above-mentioned highly contaminated commodities may tend to cause aflatoxicosis in humans or animals. Some of the highest values of aflatoxin contamination in 2015–2020 are shown in Table 3.

**Figure 1:**
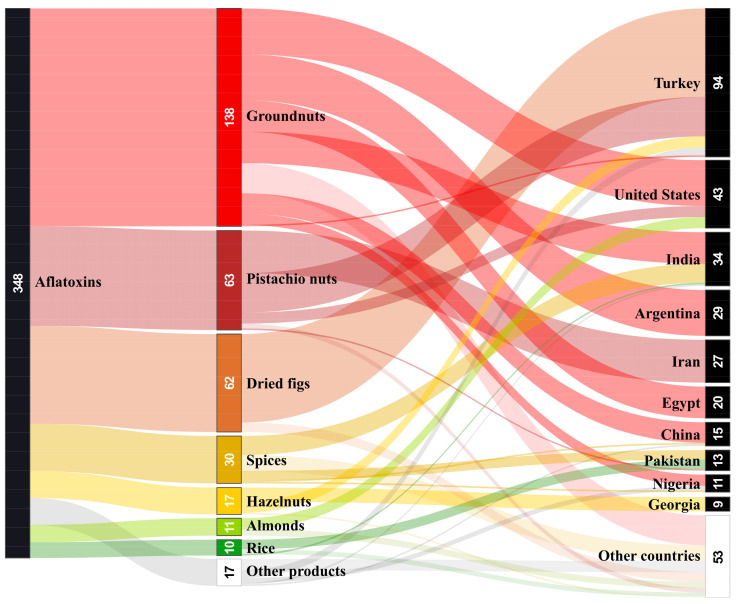
Aflatoxin notifications in food by the RASFF in 2020. Note: All products in the “Other products” category were notified less than four times in 2020. The category “Other countries” includes notifications from 24 countries, in which less than 9 notifications originated and 2 notifications were of unknown origin. Processed according to RASFF database [144].

In the year 2020, groundnuts, pistachio nuts, dried figs, spices, hazelnuts, almonds, and rice were the most notified products in relation to AF contamination. The other notified products were mostly various seeds (melon, ogbono, sunflower, lotus, and sesame seeds) and flours (wheat flour, chestnut flour, and banku mix). Single notifications concerned Brazil nuts, apricot kernels, soya, milk, and date syrup. Most notifications originated in Turkey (mainly dried figs and pistachio nuts), followed far behind by the United States (mainly groundnuts) and India (mainly groundnuts and spices). A significant number of notifications originated in Argentina (groundnuts only), Iran (pistachio nuts only), Egypt (groundnuts only), China (mainly groundnuts), Pakistan (mainly spices and rice), Nigeria (mainly groundnuts), and Georgia (hazelnuts only) (see Figure 1) [144]. Fewer notifications (the number is given in brackets) originated in other countries: Spain (7); Sri Lanka (6); Brazil (5); Italy and Ghana (3); Ethiopia, United Kingdom, Germany, Ukraine, and Cameroon (2); and Angola, Vietnam, Hong Kong, South Africa, Jordan, Togo, Hungary, Nepal, Bolivia, Cambodia, Paraguay, Indonesia, Belgium, Malaysia, Tunisia, Senegal, and Azerbaijan (1). Two notifications were of unknown origin [144].

The amount of the world production of these commodities should be taken into consideration as demonstrated in Table 4. Although groundnuts are the most often notified product, pistachio nuts can be labelled as the relatively most frequently notified product, with approximately one notification per 16,787 tons produced. In contrast, there is one notification per 344,886 tons of groundnuts produced [144].

## 4. Conclusions

The year 2020 has already passed 60 years of AF discovery. Since then, despite the scientific progress in the knowledge on AFs and the efforts made to reduce the risk they pose to public health, developing countries still have to tolerate a high level of AF contamination of foods to not compromise the food supply. Selected research topics concerning AFs continue to draw attention worldwide, such as research on the diversity and genetic variability of AF production in *Aspergillus flavus* and other AF producers, or on the problem of using biocontrol strategies for the non-aflatoxigenic strains of *A. flavus* with the goal of the better protection of public health and the prevention of economic losses. The recent occurrence data, the recent food consumption data, and the recent toxicological data of AFs in foodstuffs are required for the assessment of the severity of AF toxicity, the estimation of human dietary exposure, and health risk assessments.

## Figures and Tables

**Table 1:** Aflatoxigenic *Aspergillus* species from *Aspergillus* section *Flavi.*

Species	AF Producer	Year of Identification	Occurrence
*A. flavus*	B_1_, B_2_	1962	Peanuts, maize, spices
*A. parasiticus*	B_1_, B_2_ G_1_, G_2_	1963	Maize, peanuts
*A. nomius*	B_1_, B_2_ G_1_, G_2_	1987	Wheat, turmeric
*A. pseudonomius*	B_1_, B_2_ G_1_, G_2_	1997	Brazil nut
*A. pseudotamarii*	B_1_, B_2_	2001	Brazil nut
*A. parvisclerotigenes*	B_1_, B_2_ G_1_, G_2_	2005	Peanuts
*A. arachidicola*	B_1_, B_2_ G_1_, G_2_	2008	Carob flour
*A. luteovirescens* ^a^	B_1_, B_2_ G_1_, G_2_	2008	Brazil nut
*A. minisclerotigenes*	B_1_, B_2_ G_1_, G_2_	2008	Peanuts, curry, red chili
*A. pseudocaelatus*	B_1_, B_2_ G_1_, G_2_	2011	Peanuts, Brazil nut
*A. togoensis*	B_1_, B_2_	2011	Fruit of Landolphia spp.
*A. mottae*	B_1_, B_2_ G_1_, G_2_	2012	Maize
*A. novoparasiticus*	B_1_, B_2_ G_1_, G_2_	2012	No occurrence in food ^b^
*A. sergii*	B_1_, B_2_ G_1_, G_2_	2012	Almond
*A. transmontanensis*	B_1_, B_2_ G_1_, G_2_	2012	Almond
*A. texensis*	B_1_, B_2_ G_1_, G_2_	2018	Maize
*A. aflatoxiformans*	B_1_, B_2_ G_1_, G_2_	2019	Peanuts, sesame
*A. austwickii*	B_1_, B_2_ G_1_, G_2_	2019	Rice, sesame
*A. cerealis*	B_1_, B_2_ G_1_, G_2_	2019	Rice, maize, peanut
*A. pipericola*	B_1_, B_2_ G_1_, G_2_	2019	Black pepper
*A. agricola sp. nov.*	B_1_, B_2_	2020	Maize
*A. toxicus sp. nov.*	B_1_, B_2_	2020	Maize

^a^ Formerly named *Aspergillus bombycis*; ^b^ Sputum of leukemic patient.

**Table 2:** The share of aflatoxin notifications in 2015-2020.

Substance/Year	2015	2016	2017	2018	2019	2020
Mycotoxins	495	549	579	655	584	423
AFs ^a^	441 (89.1%)	478 (87.1%)	539 (93.0%)	567 (86.6%)	497 (85.1%)	370 (87.5%)
AFs in food	423 (95.9%)	461 (96.4%)	515 (95.5%)	510 (89.9%)	467 (94.0%)	348 (94.1%)

^a^ AFs = aflatoxins; processed according to the Rapid Alert System for Food and Feed (RASFF) database [143].

**Table 3:** The highest concentrations of aflatoxin B_1_ and total aflatoxins in foods notified by RASFF in 2015–2020.

No.	Product	AFB_1_ (µg/kg)	AFT ^a^ (µg/kg)	Origin	Year
1	Peanut paste	707,000	907,000	Senegal	2016
2	Peanuts	180,200	220,900	China	2015
3	Groundnuts in shell	42,100	46,800	Egypt	2019
4	Groundnuts	17,000	38,000	Turkey	2016
5	Pistachios	−	26,300	Germany	2020
6	Peanut in shell	24,000	26,000	China	2015
7	Almonds	−	24,000	US	2018
8	Dried figs	15,300	−	Turkey	2020
9	Roasted chopped hazelnuts	4000	15,200	Turkey	2015
10	Shelled nuts	12,890	14,420	Turkey	2019
11	Organic groundnut kernels	11,000	14,000	Egypt	2020
12	Dried red chilies	13,700	14,000	India	2020
13	Roasted and salted watermelon seeds	13,700	−	Turkey	2020
14	Shelled almonds	10,440	11,420	US	2019
15	Hazelnut kernels	7200	−	Georgia	2019

^a^ AFT = sum of aflatoxins B1, B2, G1, and G2; processed according to the RASFF database [144].

**Table 4:** The number of RASFF aflatoxin notifications concerning certain food products in relation to their average world production.

Product	Average Annual Production (2015–2019) ^a^ (Tons)	Number of Notifications by RASFF (2020)	Tons Produced per RASFF Notification
Groundnuts	47,591,548	138	344,866
Pistachio nuts	1,057,587	63	16,787
Dried figs	1,185,768	62	19,125
Spices	14,541,902	30	484,730
Hazelnuts	939,927	17	55,290
Almonds	3,039,020	11	276,275
Rice	748,304,354	10	74,830,435

^a^ Average annual spice production includes these categories: “Anise, badian, fennel, coriander”, “Chilies and peppers, dry”, “Cinnamon”, “Cloves”, “Ginger”, “Nutmeg, mace, cardamoms”, “Mustard seed”, “Pepper, *Piper* pp.”, “Peppermint”, “Vanilla”, and “Spice not elsewhere specified”. Processed according to FAOSTAT and RASFF databases [98,144].

## Data Availability

Not applicable.
